# Vertebral Fractures Beyond Bone Density in Breast Cancer: A Real-World Study of Endocrine Therapy and FRAX Reclassification

**DOI:** 10.3390/jcm15134905

**Published:** 2026-06-24

**Authors:** Réka Kollár, Tamás Leel-Őssy, Eszter Szigeti, Magdolna Dank, Éva Hosszú, Csaba Horváth, Szilvia Mészáros

**Affiliations:** 1 Department of Internal Medicine and Oncology, Semmelweis University, 1083 Budapest, Hungary; kollar.reka@semmelweis.hu (R.K.); leel.ossy.tamas@semmelweis.hu (T.L.-Ő.); eszter2szigeti@gmail.com (E.S.); dank.magdolna@semmelweis.hu (M.D.); meszaros.szilvia@semmelweis.hu (S.M.); 2 National Institute of Oncology, Comprehensive Cancer Center, 1122 Budapest, Hungary; 3 Pediatric Center, Semmelweis University, 1094 Budapest, Hungary; hosszu.eva@semmelweis.hu

**Keywords:** breast cancer, endocrine therapy, aromatase inhibitors, vertebral fractures, DXA, vertebral fracture assessment, FRAX

## Abstract

**Background**: Endocrine therapy for hormone receptor-positive breast cancer is associated with accelerated bone loss and increased fracture risk. Vertebral fractures (VFs) are frequently asymptomatic and remain underdiagnosed, potentially leading to underestimation of fracture risk. **Methods**: We conducted a cross-sectional real-world study that included 172 women with breast cancer (mean age 58.2 ± 12.0 years), the majority receiving aromatase inhibitors. Vertebral fractures were assessed using vertebral fracture assessment (VFA) during dual-energy X-ray absorptiometry (DXA). Bone mineral density (BMD), trabecular bone score (TBS), quantitative ultrasound (QUS), and FRAX^®^ scores were evaluated. **Results**: Vertebral fractures were identified in 13% of patients, and 78% of these occurred in women with normal or osteopenic BMD. Age was independently associated with VFs, while conventional densitometric and non-densitometric parameters showed limited discriminatory ability. The incorporation of VFA-detected fractures into FRAX significantly increased estimated fracture risk (hip fracture risk: 0.8% vs. 4.1%, *p* = 0.008). **Conclusions:** Vertebral fractures are common and frequently unrecognized in women receiving endocrine therapy and are not adequately captured by BMD. Routine use of VFA during DXA substantially improves fracture risk assessment and leads to a clinically meaningful reclassification of FRAX estimates. These findings support a more comprehensive approach to skeletal risk evaluation in this population.

## 1. Introduction

Breast cancer is the most common malignancy among women worldwide. In hormone receptor-positive disease, endocrine therapy—including aromatase inhibitors and selective estrogen receptor modulators—represents a cornerstone of treatment and has significantly improved survival outcomes. However, these therapies are associated with adverse skeletal effects, including accelerated bone loss and an increased risk of fragility fractures [1].

Aromatase inhibitor-induced estrogen depletion plays a central role in bone deterioration, leading to increased bone turnover and reduced bone strength. While bone mineral density (BMD) is widely used to assess fracture risk, it does not fully capture bone quality or structural integrity [2,3,4]. Consequently, a substantial proportion of fractures occur in individuals with normal or only mildly reduced BMD [5].

Vertebral fractures are among the most common osteoporotic fractures and are frequently asymptomatic. As a result, they often remain undiagnosed in routine clinical practice [6,7,8]. Their presence, however, is a strong independent predictor of future fractures and significantly influences fracture risk estimation tools such as FRAX^®^.

Vertebral fracture assessment (VFA), performed during dual-energy X-ray absorptiometry (DXA), provides a convenient and low-radiation method for detecting vertebral deformities. Despite its availability, VFA is not routinely performed in all patients at risk.

However, real-world data on the prevalence of asymptomatic vertebral fractures and their impact on FRAX-based risk stratification in women undergoing endocrine therapy remain limited.

The aim of this study was to assess the prevalence of vertebral fractures in women with breast cancer receiving endocrine therapy and to evaluate their clinical impact, particularly with respect to fracture risk estimation using FRAX.

## 2. Materials and Methods

### 2.1. Study Population

This cross-sectional study enrolled 408 pre- and postmenopausal hormone receptor-positive breast cancer female patients treated with endocrine hormone therapy.

**Figure 1:**
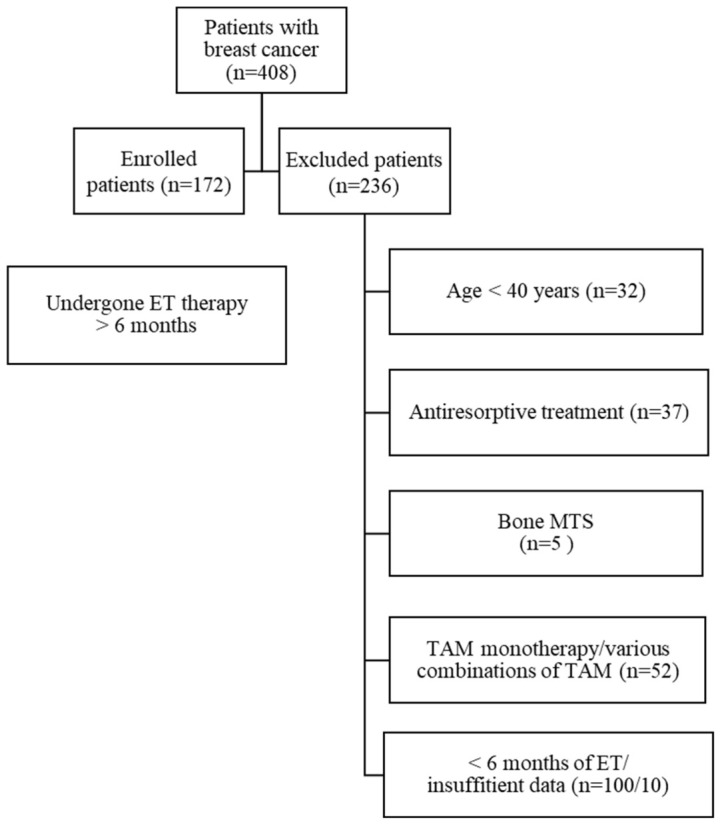
Flow chart of study participants. Abbreviations: TAM, Tamoxifen; ET, Endocrine hormone therapy; MTS, metastasis.

Inclusion criteria required patients to have undergone endocrine therapy for at least six months. Exclusion criteria included: (1) age younger than 40 years, (2) antiresorptive treatment, (3) bone metastasis, (4) Tamoxifen monotherapy or various combinations of Tamoxifen (TAM), and (5) had not received endocrine therapy, had less than six months of endocrine treatment or insufficient data (Figure 1). Patients receiving tamoxifen monotherapy or tamoxifen-containing regimens were excluded due to the distinct and potentially bone-protective effects of tamoxifen on skeletal metabolism, which could confound the assessment of aromatase inhibitor-associated bone loss and vertebral fracture risk.

A total of 236 subjects were excluded based on these criteria, leaving 172 patients for analysis. A total of 166 women in the cohort were treated with aromatase inhibitors, whereas 6 women received Gonadotropin-Releasing Hormone (GnRH) antagonist treatment.

### 2.2. Bone Assessment

Bone mineral density for each participant was determined by dual-energy X-ray absorptiometry (GE Lunar Prodigy, Madison, WI, USA) by the same trained operator and in accordance with the manufacturer’s instructions. BMD was determined at the lumbar spine (L1–L4), femoral neck, total hip and the radius. BMD was classified as normal, low, or osteoporosis according to the WHO criteria [9].

Vertebral fracture assessment was performed using the morphometric software of the GE Lunar Prodigy (Lunar Corporation, Madison, WI, USA). Vertebral morphometry is a quantitative method for assessing vertebral heights. The method determines the anterior, middle and posterior heights of each vertebra and then classifies the type and severity of vertebral fracture using the Genant classification system [10]. VFA was performed by a single experienced operator.

Trabecular bone score (TBS) was evaluated at the L1–L4 vertebral bodies using TBS iNsight software (version 3.1, MediMaps Group, Geneva, Switzerland) installed on the DXA machine (GE Lunar Prodigy, Madison, WI, USA).

Quantitative ultrasound (QUS) of the left heel was conducted in all participants using the Sahara Clinical Sonometer (Hologic, Inc., Bedford, MA, USA), following a standardized protocol across all study centers. The measurements obtained included broadband ultrasound attenuation (BUA, expressed in dB/MHz), speed of sound (SOS, in m/s), and the quantitative ultrasound index (QUI), a composite parameter calculated from SOS and BUA using the formula: QUI = 0.41 × SOS + 0.41 × BUA − 571.

All skeletal assessments were performed at a single study visit. The time since initiation of endocrine therapy was recorded for each participant to characterize treatment exposure. This parameter reflected the interval between treatment initiation and skeletal assessment and should not be interpreted as a longitudinal follow-up period. No serial assessments of bone parameters or fracture incidence were performed.

### 2.3. Fracture Risk Assessment

Fracture risk was estimated using the FRAX^®^ tool, with and without inclusion of vertebral fracture status. FRAX scores were initially calculated without including vertebral fracture status. In patients in whom previously undiagnosed vertebral fractures were identified by vertebral fracture assessment (VFA), FRAX was subsequently recalculated by entering “previous fragility fracture” as a clinical risk factor, in accordance with the FRAX^®^ tool guidance. Thus, VFA-detected vertebral fractures were incorporated into the FRAX model as a dichotomous variable (fracture present vs. absent), allowing assessment of their impact on estimated 10-year fracture risk.

### 2.4. Statistical Analysis

Data were described using descriptive statistics and presented as numbers, percentages, means, and standard deviations (SD). The prevalence of fractures among the normal, osteopenic, and osteoporotic groups was compared using the Pearson chi-square test. Continuous variables were reported as means and assessed for normality before analysis. Two-tailed *t* tests were used to assess differences in normally distributed continuous demographic variables between the groups. Statistical significance was set at *p* < 0.05.

The association between vertebral fractures and clinical variables was evaluated using logistic regression analysis. Variables with a *p*-value < 0.25 in univariate logistic regression analyses were entered into the multivariable logistic regression model. The final multivariable model included age, body mass index, aromatase inhibitor therapy, and gonadotropin-releasing hormone agonist therapy. Adjusted odds ratios (ORs) with 95% confidence intervals (CIs) were calculated using logistic regression.

Bone-related parameters, including bone mineral density, trabecular bone score, FRAX score, and prior fragility fracture, were not included in the primary multivariable model due to lack of independent contribution in univariate screening and potential overlap in the information captured by these measures. To assess the robustness of the findings, sensitivity analyses including these variables were also performed.

## 3. Results

The median age of the 172 enrolled patients was 58.2 ± 12 years (range: 40.3–88.2 years). In the overall study population, time since initiation of endocrine therapy was 49.3 ± 40.5 months (range: 6–204 months). The mean BMI was 26.9 ± 4.9 kg/m^2^ (range: 17.44–40.23 kg/m^2^).

The mean (±SD) bone mineral density (BMD) and T-scores in the lumbar, femoral neck, and radius regions were measured as follows: lumbar BMD 1.116 ± 0.18 g/cm^2^, lumbar T-score −0.7 ± 1.5, femoral BMD 0.877 ± 0.13 g/cm^2^, femoral T-score −0.8 ± 1.1, and radius BMD 0.786 ± 0.11 g/cm^2^, radius T-score −1.0 ± 1.2. Osteoporosis was diagnosed in 36 (21%) patients, while osteopenia was found in 58 (33.7%) subjects.

The mean FRAX for major osteoporotic (MOP) and hip fractures (HIP) was 6.1 ± 5.3% and 1.9 ± 3.1%, respectively. The trabecular bone score (TBS) in the entire subject population was 1.376 ± 0.13 SD, and the TBS T-score was −1.1 ± 1.3 SD. Quantitative heel ultrasound measurements included SOS, BUA, QUI, and QUS T-score, which were 1549.9 ± 36.7 m/s, 108.7 ± 15.3 dB/MHz, 86.7 ± 17.4, and −0.9 ± 1.4 SD, respectively.

The mean duration of aromatase inhibitor therapy was 26.9 ± 30.0 months (range 6–120 months), while gonadotropin-releasing hormone (GnRH) agonist therapy ranged from 6 to 88 months.

**Figure 2:**
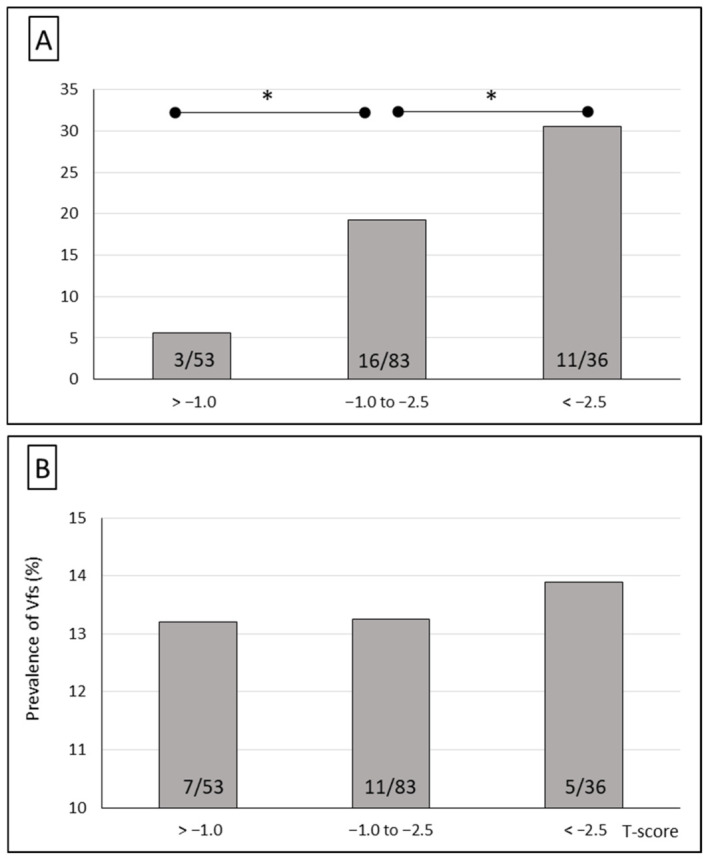
Prevalence of all fractures (**A**) and vertebral fractures (VFs) (**B**) based on normal, osteopenic, and osteoporotic T-scores. *: *p* < 0.05.

Based on anamnestic data, 30 patients (17%) had a previous low-trauma fracture, including 2 patients with a known vertebral fracture. Vertebral fracture assessment identified previously undiagnosed vertebral fractures in 13% (*n* = 23). Based on the Genant semiquantitative classification, 15 vertebral fractures were graded as Grade 2 (moderate) and 8 as Grade 3 (severe). Notably, 78% of these fractures occurred in women with normal or osteopenic BMD, indicating that fracture occurrence was not confined to those with densitometric osteoporosis (Figure 2).

Patients with fractures were significantly older compared to those without fractures (Table 1).

There was no significant association between endocrine therapy duration and vertebral fracture prevalence (OR 1.01, 95% CI 0.99–1.03, *p* = 0.27).

In multivariate analysis, age was independently associated with the presence of vertebral fractures (Table 2). Although aromatase inhibitor therapy showed an association with vertebral fractures, this finding should be interpreted with caution due to the high proportion of patients receiving this treatment in the study cohort (Table 2), as this markedly limits the variability of exposure and therefore the reliability of estimating its independent effect.

Sensitivity analyses, including bone-related parameters (BMD, TBS, FRAX score, and prior fracture), yielded results consistent with the primary model, with no relevant change in the identified predictors (see Supplementary Table S1).

The mean age was significantly higher in patients with vertebral fractures compared to those without vertebral fractures (65.3 ± 10.7 vs. 57.1 ± 11.8 years, *p* = 0.009). No significant differences were observed between patients with and without vertebral fractures in terms of BMD, TBS, QUS, or baseline FRAX scores (Table 3).

Incorporation of VFA-detected vertebral fractures into FRAX calculations resulted in a significant increase in estimated fracture risk. For example, estimated hip fracture risk increased from 0.8% to 4.1% (*p* = 0.008), indicating clinically meaningful risk reclassification (Table 4).

Among the 23 patients with previously unrecognized vertebral fractures detected by VFA, the number classified above the predefined FRAX intervention threshold increased from 4 to 8 following inclusion of vertebral fracture status in the FRAX calculation, resulting in reclassification of 4 patients (17.4%) into a higher risk category. No downward reclassification was observed.

## 4. Discussion

In this real-world study of women with breast cancer receiving endocrine therapy, vertebral fractures were common and frequently occurred in patients without densitometric osteoporosis.

These findings highlight the limitations of relying on BMD alone for fracture risk assessment in this population.

The high prevalence of vertebral fractures detected by VFA underscores the clinical importance of systematic imaging, particularly given that most vertebral fractures are asymptomatic and would otherwise remain undiagnosed [6,7,8].

Our findings are consistent with previous studies demonstrating that fractures frequently occur in individuals with normal or osteopenic BMD, reflecting the contribution of bone quality and microarchitectural deterioration beyond bone mass alone [11,12].

Endocrine hormone therapy, especially aromatase inhibitors, plays an essential role in the treatment of hormone receptor-positive breast cancer in women. The induced estrogen deficiency disrupts the physiological balance between osteoblasts and osteoclasts, leading to increased bone resorption [1,13]. The estrogen depletion induced by aromatase inhibitors can lead to bone loss and deterioration of bone microarchitecture, thus osteoporosis and fractures are recognizable and undesirable outcomes of adjuvant hormone therapies for breast cancer [1,14].

In patients receiving aromatase inhibitor therapy, bone density significantly decreased during 1 and 2 years of follow-up: lumbar BMD decreased by 2.6% and then 4%, and femoral neck BMD by 1.7% and 3.2%, respectively [15]. Further studies have shown that breast cancer patients treated with aromatase inhibitors experience bone loss that is 2- to 4-fold greater than the physiological rate in the menopause [1,13].

Estrogen deficiency contributes to bone loss by directly affecting bone cells and altering the bone remodeling process. Normally, bone resorption by osteoclasts is followed by bone formation by osteoblasts. In the absence of estrogen, the production of pro-inflammatory cytokines—such as IL-1, IL-6, IL-11, IL-17, TNF-α, RANKL, and GM-CSF—increases, promoting osteoclast activation and prolonging osteoclast survival, resulting in accelerated bone resorption. Prolonged estrogen deficiency also impairs osteoblast survival, further shifting the balance towards bone loss [16]. Consequently, chronic estrogen deficiency is marked by increased osteoclast activity and reduced osteoblast function, leading to net bone loss.

Estrogen deficiency also alters bone microarchitecture through changes in bone turnover, too. It reduces trabecular number, increases the spacing between trabeculae, transforms trabecular plates into rods, and impairs trabecular connectivity. These structural changes weaken bone quality and strength, particularly in the lumbar spine and femoral neck [16].

While aromatase inhibitor therapy is known to adversely affect bone health through estrogen depletion and increased bone turnover, its independent association with vertebral fractures could not be robustly established in this cohort due to limited variability in treatment exposure [13,17,18].

The main determinant of bone fragility is bone mineral density, while fragility can also be influenced by bone microarchitecture and other tissue properties not captured by DXA. These characteristics contribute to bone resistance to fracture. Quality assessment of bone can be challenging both in clinical practice and in research. While several techniques, such as high-resolution peripheral computed tomography (HR-pQCT), exist for research, only two indirect methods are available in routine clinical practice: the trabecular bone score and quantitative ultrasound. Trabecular bone score offers indirect information about bone microarchitecture, as it relates to specific features such as the trabecular separation, trabecular number, and connectivity density. QUS contains mixed information concerning the mass of bone plus some aspects of bone quality (e.g., elasticity) [19,20,21].

An important finding of the present study is that the conventional methods, such as BMD, TBS, QUS, and baseline FRAX scores, showed limited ability to discriminate between patients with and without vertebral fractures. This suggests that reliance on these parameters alone may lead to underestimation of fracture risk.

The prevalence of vertebral fractures observed in our cohort (13%) is consistent with previous reports demonstrating a substantial burden of skeletal fragility among women receiving aromatase inhibitor therapy. However, reported prevalence rates vary considerably according to study design and fracture ascertainment methods [22]. In particular, studies based on clinically recognized fractures tend to report lower rates than those incorporating systematic vertebral imaging.

Large randomized trials, including the ATAC and BIG 1–98 studies, primarily recorded clinically apparent fractures and did not systematically assess asymptomatic vertebral fractures. In the ATAC trial, vertebral fractures were reported in approximately 5% of women receiving anastrozole compared with 1% of those treated with tamoxifen [15]. Similarly, in the BIG 1–98 study, vertebral fracture rates were 1.1% in women receiving letrozole and 0.9% in those receiving tamoxifen [3]. These relatively low prevalences likely reflect the under-recognition of clinically silent vertebral fractures, which are known to account for a substantial proportion of all vertebral fractures in postmenopausal women.

By contrast, studies employing dedicated vertebral imaging have demonstrated a markedly higher prevalence of vertebral fractures. Mazziotti et al. evaluated 413 women with breast cancer receiving endocrine therapy and identified vertebral fractures in 22.1% of patients using vertebral fracture assessment performed during DXA examinations [2]. Notably, vertebral fractures were observed across the entire spectrum of bone mineral density categories and were not restricted to women with densitometric osteoporosis. The higher prevalence reported by Mazziotti et al. may partly reflect differences in patient characteristics and treatment exposure; however, it also highlights the superior sensitivity of systematic VFA-based screening for detecting clinically unrecognized vertebral fractures. As in their study, the majority of vertebral fractures in our cohort occurred in women with normal or osteopenic BMD, with 78% of fractures identified outside the osteoporotic range. These findings reinforce the concept that bone mineral density alone does not fully capture skeletal fragility in women receiving endocrine therapy.

The prevalence observed in the present study lies between that reported in large clinical trials and that reported in cohorts undergoing systematic vertebral imaging. This finding supports the view that the true burden of vertebral fractures among women treated with aromatase inhibitors is likely underestimated when fracture assessment relies solely on clinical presentation. The routine incorporation of VFA into DXA examinations may therefore provide clinically relevant information that is not captured by conventional fracture history or bone mineral density measurements alone, ultimately improving fracture risk stratification and clinical decision-making [2].

Recent international guidelines on cancer treatment–induced bone loss, including updated recommendations [23], emphasize the importance of early identification of patients at increased fracture risk during aromatase inhibitor therapy. Although bone mineral density remains the cornerstone of assessment, these guidelines increasingly acknowledge the added value of detecting vertebral fractures, particularly given their frequent asymptomatic nature and strong prognostic relevance. Vertebral fracture assessment (VFA), performed alongside DXA, is recommended in selected high-risk individuals to improve fracture risk stratification and guide treatment decisions. However, its implementation in routine clinical practice remains variable across centers. Our findings support these recommendations by demonstrating a substantial prevalence of previously unrecognized vertebral fractures and clinically meaningful reclassification of fracture risk when VFA findings are incorporated into FRAX-based assessment.

The inclusion of VFA-detected silent vertebral fractures in FRAX calculations resulted in a clinically relevant reclassification of fracture risk at the individual patient level. These findings highlight the potential value of identifying previously unrecognized vertebral fractures in risk assessment and support the role of VFA in improving fracture risk stratification in this population.

## 5. Limitations

This study has several limitations. Its cross-sectional design precludes causal inference. A further potential limitation is the selection bias introduced by the exclusion of a substantial proportion of initially screened patients, which may limit the generalizability of the findings to the broader population of women with breast cancer receiving endocrine therapy.

The predominance of aromatase inhibitor use limits the ability to compare treatment subgroups and restricts the interpretation of AI-specific effects. As patients receiving tamoxifen-containing regimens were excluded, the findings are primarily applicable to AI-treated patients and may not be generalizable to the broader endocrine-treated breast cancer population. Therefore, the observed association between AI therapy and vertebral fractures should be interpreted with caution, as the limited variability in exposure reduces the reliability of estimating independent treatment effects and does not exclude residual confounding. In addition, the marked imbalance in treatment exposure (166/172 patients receiving aromatase inhibitors) further limits the robustness of any inference regarding AI therapy as an independent predictor.

A number of clinically relevant variables that may influence bone density and fracture risk, including menopausal status, exposure to chemotherapy and radiotherapy, smoking status, corticosteroid use, vitamin D status, and calcium supplementation, were not systematically assessed in the present study due to its retrospective and cross-sectional design and incomplete data availability. These factors may act as potential confounders and should be considered when interpreting the findings.

The relatively small number of vertebral fracture events identified by VFA represents an important limitation of this study, as it reduces the statistical power of subgroup and multivariable analyses. The limited number of outcome events (*n* = 23) may affect the stability of multivariable logistic regression estimates and may increase the risk of model overfitting. However, sensitivity analyses including additional bone-related covariates yielded consistent results, supporting the robustness of the findings. In particular, the events-per-variable ratio in the logistic regression model was at the lower acceptable threshold, and therefore the possibility of model overfitting cannot be excluded. Consequently, the detection of modest associations between vertebral fractures and skeletal parameters, such as TBS, may have been limited. This may partly explain why TBS showed a significant association with overall fracture prevalence, whereas no statistically significant association was observed when the analysis was restricted to vertebral fractures alone. Given the limited number of events, the predictive findings derived from the multivariable models should also be interpreted with caution.

Additionally, the sample size, while adequate for exploratory analysis, may limit generalizability.

## 6. Clinical Implications

Our findings support the integration of vertebral fracture assessment into routine DXA evaluation in women with breast cancer receiving endocrine therapy. This approach may improve fracture risk stratification and optimize preventive strategies.

## 7. Conclusions

Vertebral fractures are common and frequently unrecognized in women with breast cancer receiving endocrine therapy, and their occurrence is not adequately explained by bone mineral density alone. Incorporating vertebral fracture assessment into routine DXA evaluation enables improved detection of clinically silent fractures and leads to meaningful changes in FRAX-based risk estimation. These findings support the routine use of VFA in this population to enhance fracture risk stratification and guide preventive strategies.

## Figures and Tables

**Table 1:** Characteristics of fractured (non-vertebral + vertebral) vs. non-fractured patients.

Variable	Fractured (*n* = 56)	Non-Fractured (*n* = 116)	*p*
Age (years)	65.5 (10.6)	56.7 (11.8)	<0.001
BMI (kg/m^2^)	26.7 (4.1)	26.2 (5.0)	0.546
Time since initiation of endocrine therapy (months)	42.9 (35.4)	50.7 (41.7)	0.294
LS BMD g/cm^2^	1.088 (0.24)	1.121 (0.16)	0.472
FN BMD g/cm^2^	0.850 (0.13)	0.883 (0.13)	0.223
R BMD g/cm^2^	0.880 (0.13)	0.926 (0.13)	0.088
TBS	1.324 (0.12)	1.384 (0.11)	0.015
SOS (m/s)	1535.9 (45.5)	1553.4 (33.6)	0.142
BUA (dB/MHz)	106.1 (21.6)	109.3 (13.5)	0.547
QUI	81.5 (20.9)	87.4 (16.3)	0.282
FRAX HIP (%)	3.5 (3.6)	1.6 (2.8)	<0.001
FRAX MOP (%)	11.9 (5.7)	4.9 (4.4)	0.002

Abbreviations: BMI, body mass index; BMD, bone mineral density; LS, lumbar spine; FN, femoral neck; R, radius; TBS, trabecular bone score; SOS, speed of sound; BUA, broadband ultrasound attenuation; QUI, Quantitative Ultrasound Index; MOP, major osteoporotic fractures.

**Table 2:** Multivariate analysis of the association of multiple variables with vertebral fractures.

Variable	β Coefficient	Standard Error	Wald χ^2^	Adjusted OR (95%CI)	*p* Value
Age	0.086	0.037	5.4	1.09 (1.03–1.15)	0.020
BMI	−0.062	0.041	2.28	0.94 (0.86–1.03)	0.130
AI therapy	0.908	0.353	6.61	2.48 (1.10–5.60)	0.010
GnRH agonist therapy	0.140	0.365	0.15	1.15 (0.56–2.37)	0.70

Abbreviations: BMI, body mass index; AI: Aromatase Inhibitor; GnRH: Gonadotropin-releasing hormone.

**Table 3:** Clinical characteristics of patient with VFs women, and patient without VFs women [mean (SD)].

Variable	Patients with VFs (*n* = 23)	Patients Without VFs (*n* = 149)	*p*
Age (years)	65.3 (10.7)	57.1 (11.8)	0.009
BMI (kg/m^2^)	24.9 (4.5)	26.2 (4.8)	0.150
Time since initiation of endocrine therapy (months)	43.1 (34.8)	50.3 (40.6)	0.380
LS BMD g/cm^2^	1.007 (0.18)	1.110 (0.17)	0.260
FN BMD g/cm^2^	0.840 (0.13)	0.888 (0.13)	0.180
R BMD g/cm^2^	0.775 (0.11)	0.781 (0.11)	0.410
TBS	1.346 (0.1)	1.356 (0.12)	0.120
SOS (m/s)	1542.7 (53.5)	1551.3 (38.0)	0.310
BUA (dB/MHz)	103.2 (18.4)	109.1 (14.5)	0.177
QUI	83.4 (17.5)	86.9 (17.4)	0.350
FRAX HIP (%)	2.8 (2.7)	1.8 (2.6)	0.101
FRAX MOP (%)	8.3 (6.3)	5.9 (4.8)	0.099

Abbreviations: VFs, vertebral fracture; BMI, body mass index; BMD, bone mineral density; LS, lumbar spine; FN, femoral neck; R, radius; TBS, trabecular bone score; SOS, speed of sound; BUA, broadband ultrasound attenuation; QUI, Quantitative Ultrasound Index; MOP, major osteoporotic fractures.

**Table 4:** FRAX-estimated fracture risk by VFA status.

	Without VFA Fracture	With VFA Fracture	*p*
FRAX HIP (%)	0.8 (1.4)	4.1 (3.5)	0.008
FRAX MOP (%)	7.6 (7.3)	9.3 (7.6)	<0.001

Abbreviations: VFA, vertebral fracture assessment; MOP, major osteoporotic fractures.

## Data Availability

The original contributions presented in this study are included in the article. Further inquiries can be directed to the corresponding author.

## Supplementary Materials

The following supporting information can be downloaded at: https://www.mdpi.com/article/10.3390/jcm15134905/s1, Table S1: Sensitivity analysis: extended multivariable logistic regression model for vertebral fractures.

## References

[1] Ye C., Leslie W.D. (2023). Fracture risk and assessment in adults with cancer. Osteoporos. Int..

[2] Mazziotti G., Vena W., Pedersini R., Piccini S., Morenghi E., Cosentini D., Zucali P., Torrisi R., Sporeni S., Simoncini E.L. (2022). Prediction of vertebral fractures in cancer patients undergoing hormone deprivation therapies: Reliability of who fracture risk assessment tool (frax) and bone mineral density in real-life clinical practice. J. Bone Oncol..

[3] Rabaglio M., Sun Z., Price K.N., Castiglione-Gertsch M., Hawle H., Thürlimann B., Mouridsen H., Campone M., Forbes J.F., Paridaens R.J. (2009). Bone fractures among postmenopausal patients with endocrine-responsive early breast cancer treated with 5 years of letrozole or tamoxifen in the BIG 1-98 trial. Ann. Oncol..

[4] Bouvard B., Hoppé E., Soulié P., Georgin-Mege M., Jadaud E., Abadie-Lacourtoisie S., Le Manac’h A.P., Laffitte A., Levasseur R., Audran M. (2012). High prevalence of vertebral fractures in women with breast cancer starting aromatase inhibitor therapy. Ann. Oncol..

[5] Fraenkel M., Geffen D.B., Novack V., Shafat T., Mizrakli Y., Ariad S., Koretz M., Norton L., Siris E. (2015). Breast cancer survivors are at an increased risk for osteoporotic fractures not explained by lower BMD: A retrospective analysis. npj Breast Cancer.

[6] Clark P., Cons-Molina F., Deleze M., Ragi S., Haddock L., Zanchetta J.R., Jaller J.J., Palermo L., Talavera J.O., Messina D.O. (2009). The prevalence of radiographic vertebral fractures in Latin American countries: The Latin American Vertebral Osteoporosis Study (LAVOS). Osteoporos. Int..

[7] Jackson S.A., Tenenhouse A., Robertson L. (2000). Vertebral fracture definition from population-based data: Preliminary results from the Canadian Multicenter Osteoporosis Study (CaMos). Osteoporos. Int..

[8] O’Neill T.W., Felsenberg D., Varlow J., Cooper C., Kanis J.A., Silman A.J. (1996). The prevalence of vertebral deformity in European men and women: The European Vertebral Osteoporosis Study. J. Bone Miner. Res..

[9] Kanis J.A. (1994). Assessment of fracture risk and its application to screening for postmenopausal osteoporosis: Synopsis of a WHO report. Osteoporos. Int..

[10] Genant H.K., Wu C.Y., van Kuijk C., Nevitt M.C. (1993). Vertebral fracture assessment using a semiquantitative technique. J. Bone Miner. Res..

[11] Nguyen N.D., Eisman J.A., Center J.R., Nguyen T.V. (2007). Risk factors for fracture in nonosteoporotic men and women. J. Clin. Endocrinol. Metab..

[12] Kadri A., Binkley N., Daffner S.D., Anderson P.A. (2022). Clinical risk factor status in patients with vertebral fracture but normal bone mineral density. Spine J..

[13] Hadji P., Aapro M., Al-Dagri N., Alokail M., Biver E., Body J.J., Brandi M.L., Brown J., Confavreux C., Cortet B. (2025). Management of aromatase inhibitor-associated bone loss (AIBL) in women with hormone-sensitive breast cancer: An updated joint position statement of the IOF, CABS, ECTS, IEG, ESCEO, IMS, and SIOG. J. Bone Oncol..

[14] Monteverdi S., Pedersini R., Gallo F., Maffezzoni F., Dalla Volta A., Di Mauro P., Turla A., Vassalli L., Ardine M., Formenti A.M. (2021). The Interaction of Lean Body Mass with Fat Body Mass Is Associated with Vertebral Fracture Prevalence in Women with Early Breast Cancer Undergoing Aromatase Inhibitor Therapy. J. Bone Miner. Res. Plus.

[15] Howell A., Cuzick J., Baum M., Buzdar A., Dowsett M., Forbes J.F., Hoctin-Boes G., Houghton J., Locker G.Y., Tobias J.S. (2005). Results of the ATAC (Arimidex, Tamoxifen, Alone or in Combination) trial after completion of 5 years’ adjuvant treatment for breast cancer. Lancet.

[16] Manolagas S.C., O’Brien C.A., Almeida M. (2013). The role of estrogen and androgen receptors in bone health and disease. Nat. Rev. Endocrinol..

[17] Cucciniello L., Garufi G., Di Rienzo R., Martinelli C., Pavone G., Giuliano M., Arpino G., Montemurro F., Del Mastro L., De Laurentiis M. (2023). Estrogen deprivation effects of endocrine therapy in breast cancer patients: Incidence, management and outcome. Cancer Treat. Rev..

[18] Christensen Holz S. (2023). Aromatase Inhibitor Musculoskeletal Syndrome and Bone Loss: A Review of the Current Literature. Curr. Oncol. Rep..

[19] Hans D., Goertzen A.L., Krieg M.A., Leslie W.D. (2011). Bone microarchitecture assessed by TBS predicts osteoporotic fractures independent of bone density: The Manitoba study. J. Bone Miner. Res..

[20] Winzenrieth R., Michelet F., Hans D. (2013). Three-dimensional (3D) microarchitecture correlations with 2D projection image gray-level variations assessed by trabecular bone score using high-resolution computed tomographic acquisitions: Effects of resolution and noise. J. Clin. Densitom..

[21] Harvey N.C., Glüer C.C., Binkley N., McCloskey E.V., Brandi M.L., Cooper C., Kendler D., Lamy O., Laslop A., Camargos B.M. (2015). Trabecular bone score (TBS) as a new complementary approach for osteoporosis evaluation in clinical practice. Bone.

[22] Diacinti D., Guglielmi G. (2010). Vertebral morphometry. Radiol. Clin. N. Am..

[23] Waqas K., Lima Ferreira J., Tsourdi E., Body J.J., Hadji P., Zillikens M.C. (2021). Updated guidance on the management of cancer treatment-induced bone loss (CTIBL) in pre- and postmenopausal women with early-stage breast cancer. J. Bone Oncol..

