# Supplementary material for: Vertebral Fractures Beyond Bone Density in Breast Cancer: A Real-World Study of Endocrine Therapy and FRAX Reclassification
Source: J Clin Med. 2026 Jun 24;15(13):4905. doi: 10.3390/jcm15134905 (PMC13361699; doi:10.3390/jcm15134905)
Supplement: Supplementary file 1 [file jcm-15-04905-s001.zip › jcm-4358112-supplementary.pdf]

Table S1. Sensitivity analysis: extended multivariable logistic regression model for vertebral fractures.

Variable	β Coefficient	SE	Wald χ^2	Adjusted OR (95% CI)	*p*-Value
Age	0.082	0.039	4.45	1.09 (1.01–1.18)	0.035
BMI	−0.058	0.043	1.82	0.94 (0.86–1.04)	0.177
AI therapy	0.742	0.401	3.42	2.10 (0.96–4.98)	0.064
GnRH agonist therapy	0.118	0.372	0.10	1.12 (0.54–2.31)	0.748
Femoral neck BMD	−0.615	0.488	1.58	0.54 (0.21–1.37)	0.209
Lumbar spine BMD	−0.402	0.436	0.85	0.67 (0.28–1.59)	0.356
TBS	−0.521	0.421	1.53	0.59 (0.26–1.34)	0.216
Prior fragility fracture	0.884	0.512	2.98	2.42 (0.89–6.55)	0.084
FRAX hip (%)	0.072	0.061	1.39	1.07 (0.95–1.21)	0.238

Abbreviations: BMI, body mass index; AI: Aromatase Inhibitor, GnRH: Gonadotropin-releasing hormone; BMD: bone mineral density; TBS: trabecular bone score.
